# Safety profile of prophylactic rescue dosing of immediate-release oral opioids in cancer patients

**DOI:** 10.1186/s40780-018-0121-3

**Published:** 2018-09-10

**Authors:** Rei Tanaka, Hiroshi Ishikawa, Tetsu Sato, Michihiro Shino, Katsuhiro Omae, Tetsumi Sato, Iwao Osaka

**Affiliations:** 1 Department of Pharmacy, Shizuoka Cancer Center, 1007 Shimonagakubo, Nagaizumi-cho, Sunto-gun, Shizuoka-ken, 411-8777 Japan; 2 Clinical Research Promotion Unit of Clinical Research Center, Shizuoka Cancer Center, 1007 Shimonagakubo, Nagaizumi-cho, Sunto-gun, Shizuoka-ken, 411-8777 Japan; 3 Department of Palliative Medicine, Shizuoka Cancer Center, 1007 Shimonagakubo, Nagaizumi-cho, Sunto-gun, Shizuoka-ken, 411-8777 Japan

**Keywords:** Opioid, Rescue dose, Immediate-release opioid, Prophylactic rescue, Morphine, Oxycodone

## Abstract

**Background:**

Appropriate prophylactic rescue dosing of opioids is considered effective for cancer pain relief, but no study has reported the safety of such prophylactic rescue. We compared the safety of prophylactic rescue dosing of immediate-release oral opioids with that of regular rescue dosing.

**Methods:**

The study included 103 cancer patients who used either immediate-release morphine syrup or immediate-release oxycodone powder at Shizuoka Cancer Center between January and December 2016. Patients were divided into those who mostly used (prophylactic group) and those who never used (regular group) prophylactic rescue doses of opioids and compared the incidence of adverse events (AEs). We also investigated whether the prophylactic rescue dose negatively interfered with its objective activity, such as meals.

**Results:**

Incidence of each AE in the prophylactic versus regular groups was as follows: somnolence, 20.6% versus 14.3%; nausea, 22.1% versus 17.1%; constipation, 19.1% versus 20.0%; urinary retention, 1.5% versus 2.9%; delirium, 4.4% versus 8.6%; and pruritus, 0% versus 2.9%. No serious AE associated with prophylactic rescue dosing was observed. No significant difference was observed in the incidence of any AE between the two groups (*p* > 0.05, Fisher’s exact test). No AE interfered with the objective activity of the prophylactic rescue dose.

**Conclusion:**

Incidence of AEs associated with prophylactic rescue dosing is not different from that associated with regular rescue dosing. In addition, the prophylactic rescue dose did not adversely affect its objective activity, suggesting the safety of appropriate prophylactic rescue dosing was similar to that of regular rescue dosing.

**Trial registration:**

The study approval number in the institution; H29-J30–29–1-3. Registered June 5, 2017.

## Background

Appropriate use of immediate-release opioid oral agents is a critical component of cancer pain relief. This is also supported by the principles of analgesics use specified in the WHO cancer pain relief program [1, 2], which state that immediate-release agents should be administered orally at individualized doses with meticulous care.

Until recently, oral opioids were only available in an immediate-release form requiring multiple daily doses. The subsequent development of the extended-release oral formulations of morphine [3], oxycodone [4], and tapentadol [5], and the fentanyl extended-release patch [6] has led to decreased daily regular dosing frequency of opioids. Improvements have also been made to immediate-release agents, such as the development of the buccal tablet [7] and sublingual tablet [8] formulations of fentanyl, which otherwise undergoes extensive first-pass metabolism. The combined use of a regular dose of an extended-release agent and a rescue dose of an immediate-release agent has become widely accepted as a standard of care. A comparison of the analgesic effect of extended-release and immediate-release agents has shown no significant difference in terms of efficacy and incidence of adverse events (AEs) [9]. It is nevertheless considered preferable to combine the two formulations for better patient adherence [10, 11].

Immediate-release agents require some time before the blood concentration of orally administered opioid starts to increase (T_max_ = 0.9 h for immediate-release morphine syrup [12] and 1.9 h for immediate-release oxycodone powder [13]). A previous study has also shown that the onset of action for the analgesic effect of an immediate-release opioid is 30 min to 1 h, as assessed by a reduction on the Numeric Rating Scale (NRS) for pain [14]. Therefore, prophylactic rescue dosing is often used in clinical practice, especially before activities that may cause breakthrough pain, such as body movement, interventions, and meals [15]. Currently, the efficacy of prophylactic rescue dosing has not yet been reported. However, recommendations from the European Association for Palliative Care (EAPC) in 2012 advocated prophylactic use of immediate-release agents, more precisely, administration of such agents 20–30 min before the predicted episode of breakthrough pain [16], and guidelines of the Japanese Society for Palliative Medicine (JSPM), published in 2014, recommended oral administration of an immediate-release opioid 30–60 min before the predicted episode of breakthrough pain when triggers are not eliminated [17]. Moreover, no study has reported the safety of prophylactic rescue dosing. Anticipated problems associated with prophylactic rescue dosing include AEs such as somnolence and nausea, which may interfere with prophylactic interventions, and increased total opioid dose, which may result in increased incidence of dose-dependent AEs, such as constipation and delirium [18, 19]. In this study, we compared the incidence of AEs associated with prophylactic versus regular rescue dosing of two easy-to-use, widely available opioids, namely, immediate-release morphine syrup and the immediate-release oxycodone powder.

## Methods

This study included 103 cancer patients hospitalized at Shizuoka Cancer Center who had started using the immediate-release morphine syrup or immediate-release oxycodone powder between January and December 2016. The study period was defined as the time from the start to discontinuation or change of treatment. Patients were divided into those who used (prophylactic group) and did not use (regular group) a prophylactic rescue dose of opioid. In this study, based on the EAPC recommendations [16] and the JSPM guidelines [17], we defined prophylactic rescue dose as a rescue dose within 1 h before an activity expected to cause breakthrough pain, such as meals, bedtime, radiation therapy, rehabilitation or intervention. The prophylactic group contained patients for whom more than half of all rescue doses were prophylactic, and the regular group contained patients for whom no rescue doses were prophylactic. The objective activities of prophylactic rescue doses were also examined in the prophylactic group.

Electronic medical records were retrospectively reviewed to extract six opioid-related AEs (somnolence, nausea, constipation, urinary retention, delirium, and pruritus) selected from those listed in the JSPM guidelines [17] based on our clinical importance, and the incidence of each AE was compared between groups. We also analyzed the incidence of serious AEs, including fall, loss of consciousness, and respiratory depression and determined which serious AE interfered with the objective activity of the prophylactic rescue dose. AEs of grade 2 or higher, according to the Common Terminology Criteria for Adverse Events (CTCAE) Ver. 4.0, were included in the analysis. Fisher’s exact test was used for statistical analysis of the AEs, with a significance level of 0.05. We also investigated whether patients used preventive medicines for AEs of prophylactic rescue, such as antiemetic drugs. Patients who had undergone chemotherapy during the study period were excluded because of the considerable influence of chemotherapy on the occurrence of AEs such as somnolence and nausea.

The two groups were compared for the following patient background factors that could potentially affect the incidence of AEs: amount of each single rescue dose, daily total rescue dose, dosing duration, dosing frequency, distribution and dose of concomitant extended-release opioids, age, sex, performance status, distribution of carcinoma (head and neck cancer, lung cancer, breast cancer, digestive system cancer, urological cancer, gynecological cancer, skin cancer, and others), renal dysfunction, and hepatic dysfunction. According to the CTCAE ver. 4.0, a grade 1 or higher elevation in serum creatinine level (≥1.04 and ≥ 0.79 mg/dL in men and women, respectively) was defined as renal dysfunction, and a grade 1 or higher elevation in serum aspartate aminotransferase/alanine transaminase level (≥40 U/L for both) was defined as hepatic dysfunction. For statistical analysis, the Mann–Whitney U test was used for the comparison of the amount of each single rescue dose, daily total rescue dose, dosing duration, dosing frequency, dose of concomitant extended-release opioids, and age. The Cochran-Armitage trend test was used for distribution of extended-release opioids and distribution of carcinoma, and Fisher’s exact test was used for the remaining factors, with a significance level of 0.05.

This study was conducted in compliance with the Ethical Guidelines for Medical Research in Humans, and with approval by the ethics committee at Shizuoka Cancer Center. The first author (R.T.) conducted all retrospective reviews of electronic medical records filled in by doctors, nurses, or pharmacists (including the first author) and statistical analyses.

## Results

**Fig. 1:**
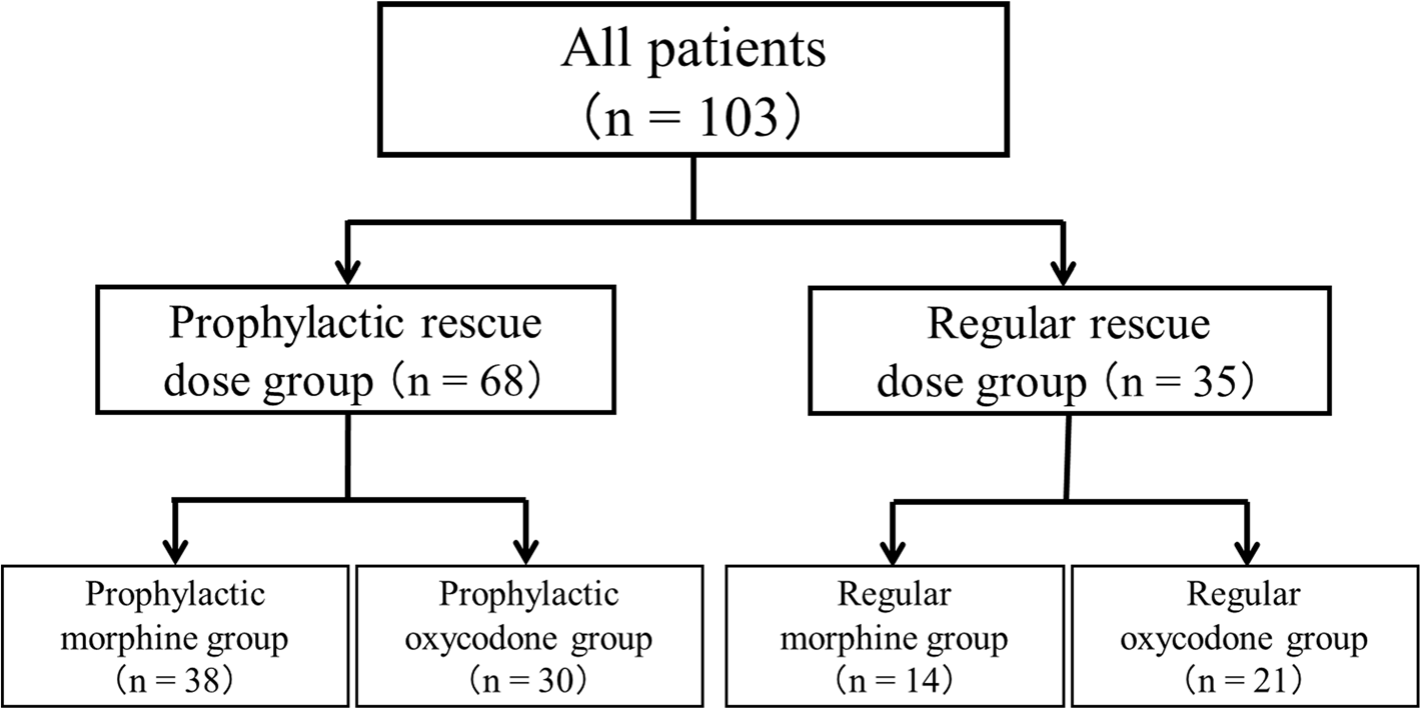
The device for quantitative analyses of perception and painful sensations and its probe. Fig. 1 indicated the 103 patients included in the study, 68 and 35 patients were in the prophylactic and regular groups, respectively. Patients were further divided into subgroups based on the type of opioid used; 38, 30, 14, and 21 patients were in the prophylactic morphine, prophylactic oxycodone, regular morphine, and regular oxycodone groups, respectively

Of the 103 patients included in the study, 68 and 35 patients were in the prophylactic and regular groups, respectively. Patients were further divided into subgroups based on the type of opioid used; 38, 30, 14, and 21 patients were in the prophylactic morphine, prophylactic oxycodone, regular morphine, and regular oxycodone groups, respectively (Fig. 1).

Concomitant extended-release opioids included 12-h extended-release oral morphine, 12-h extended-release oral oxycodone, 12-h extended-release oral tapentadol, and 1-day extended-release fentanyl patch. As shown in Table 1, the distribution of morphine was relatively high in the prophylactic group (55.9 and 39.8% in patients who received immediate-release and extended-release opioids, respectively), while that of oxycodone was relatively high in the regular group 60.0 and 54.2% in those who received immediate-release and extended-release opioids, respectively), although there were no significant differences between the two groups (P ≥ 0.05). Of all patients, 17.5% (prophylactic group: 19.1%; regular group: 14.3%) used no 12-h extended-release opioid and used only rescue-dose immediate-release opioids.

**Table 1:** Patient background

Patient background factor	Prophylactic rescue	Regular rescue	*P* value
	*n* = 68	*n* = 35	
Distribution of immediate-release opioids, n (%)			
Morphine syrup	38 (55.9)	14 (40.0)	0.15 ^a^
Oxycodone powder	30 (44.1)	21(60.0)	
Median single rescue dose (mg)^d^			
All patients	5 (3.75–15)	3.75 (3.75–15)	0.63 ^b^
Morphine syrup	5 (5–15)	5 (5–15)	0.38 ^b^
Oxycodone powder	3.75 (3.75–15)	3.75 (3.75–15)	0.32 ^b^
Median total rescue dose (mg/day)^d^			
All patients	15 (0–45)	10 (0–40)	0.21 ^b^
Morphine syrup	15 (0–40)	10 (0–40)	0.32 ^b^
Oxycodone powder	7.5 (0–45)	7.5 (0–30)	0.36 ^b^
Distribution of extended-release opioids, *n* (%)			0.17 ^c^
12-h extended-release oral morphine	27 (39.8)	8 (22.9)	
12-h extended-release oral oxycodone	24 (35.2)	19 (54.2)	
12-h extended-release oral tapentadol	3 (4.4)	1 (2.9)	
1-day extended-release fentanyl patch	1 (1.5)	2 (5.7)	
None	13 (19.1)	5 (14.3)	
Median regular dose of extended-release opioid (mg/day) ^d^	15 (0–75)	15 (0–90)	0.52^b^
Median duration of rescue dose (day)	21 (7–178)	17 (7–142)	0.51^b^
Median frequency of rescue dose (times/day)	3 (0–8)	2 (0–8)	0.30^b^
Median age (range)	69 (37–94)	69 (21–86)	0.19^b^
Sex, *n* (%)			
Men	45 (23)	22 (70.3)	0.64^a^
Women	14 (35.0)	13 (29.7)	
Performance status, *n* (%)			
≤ 2	46 (45.0)	21 (51.2)	0.66^a^
≥ 3	22 (55.0)	20 (48.8)	
Distribution of carcinoma, *n* (%)			0.43 ^c^
Head and neck cancer	17 (25.0)	9 (25.7)	
Lung cancer	18 (26.5)	7 (20.0)	
Breast cancer	3 (4.4)	2 (5.7)	
Digestive system cancer	15 (22.1)	10 (28.6)	
Urological cancer	5 (7.4)	1 (2.9)	
Gynecological cancer	3 (4.4)	3 (8.6)	
Skin cancer	2 (2.9)	1 (2.9)	
Others	5 (7.4)	2 (5.7)	
Renal dysfunction, *n* (%)	11 (25.0)	5 (36.7)	0.34^a^
Hepatic dysfunction, *n* (%)	13 (27.5)	6 (36.7)	0.48^a^

a) Fisher’s exact test

b) Mann–Whitney U test

c) Cochran-Armitage trend test

d) Converted to oral morphine equivalent doses

(oral morphine/oral oxycodone/oral tapentadol/fentanyl patch = 30:20:100:1)

No significant difference was observed between the prophylactic and regular groups for any of the patient background factors. The conversion ratios of each opioid agent were as follows: oral morphine/oral oxycodone/oral tapentadol/fentanyl patch = 30:20:100:1 [20–22].

We also investigated the purpose for which prophylactic rescue doses were used. We found that patients used a prophylactic rescue dose before meals (pain relief while eating), before bedtime (pain relief while falling asleep), before meals and bedtime (pain relief while eating and falling asleep), before radiation therapy (pain relief during radiation therapy) or before rehabilitation/intervention (pain relief during rehabilitation or intervention), and that patients decided to use a prophylactic rescue dose on their own for relief of pain, respiratory distress, or abdominal bloating. The distribution of the purposes of use is shown in Table 2.

**Table 2:** Purposes of use of prophylactic rescue doses

Purpose	Prophylactic morphine	Prophylactic oxycodone	Total
	*n* = 38	*n* = 30	*n* = 68
Before meals (pain relief while eating)	13	3	16
Before bedtime (pain relief while falling asleep)	3	10	13
Before meals and bedtime (pain relief while eating and falling asleep)	6	2	8
Before radiation therapy (pain relief during radiation therapy)	6	4	10
Before rehabilitation or intervention (pain relief during rehabilitation or intervention)	2	6	8
Patient’s decision to use for pain	3	5	8
Patient’s decision to use for respiratory distress	4	0	4
Patient’s decision to use for abdominal bloating	1	0	1

The incidence of each AE in the prophylactic versus regular groups was as follows: somnolence, 20.6% versus 14.3%; nausea, 22.1% versus 17.1%; constipation, 19.1% versus 20.0%; urinary retention, 1.5% versus 2.9%; delirium, 4.4% versus 8.6%; and pruritus, 0% versus 2.9%. Thus, no significant difference was observed in the incidence of any AE between the two groups (Table 3). Furthermore, there were no serious AEs attributable to the prophylactic rescue dose, such as fall, loss of consciousness, respiratory depression or interfering with the objective activity of the prophylactic rescue dose. None of the patients discontinued the use of opioid due to an effect of prophylactic rescue dosing. None of the patients in this study had drug dependence, such as chemical coping, or used preventive medicines for AEs of prophylactic rescue.

**Table 3:** Comparison of the incidence of adverse events between the prophylactic and regular groups in the entire study population

Adverse event	Prophylactic rescue	Regular rescue	*P* value
	*n* = 68	*n* = 35	
Somnolence, *n* (%)	14 (20.6)	5 (14.3)	0.59^a^
Nausea, *n* (%)	15 (22.1)	6 (17.1)	0.62^a^
Constipation, *n* (%)	13 (19.1)	7 (20.0)	1.00^a^
Urinary retention, *n* (%)	1 (1.5)	1 (2.9)	1.00^a^
Delirium, *n* (%)	3 (4.4)	3 (8.6)	0.40^a^
Pruritus, *n* (%)	0 (0)	1 (2.9)	0.34^a^

^a^Fisher’s exact test

Tables 4 and 5 show the results of subgroup analysis based on opioid type in the morphine and oxycodone subgroups, respectively. In both subgroups, no significant difference in AE incidence was observed between the prophylactic and regular groups.

**Table 4:** Comparison of the incidence of adverse events between the prophylactic and regular groups in the morphine subgroup

Adverse event	Prophylactic morphine	Regular morphine	*P* value
	*n* = 38	*n* = 14	
Somnolence, *n* (%)	6 (15.8)	2 (14.3)	1.00^a^
Nausea, *n* (%)	10 (26.3)	4 (28.6)	1.00^a^
Constipation, *n* (%)	12 (31.6)	4 (28.6)	1.00^a^
Urinary retention, *n* (%)	0 (0)	1 (7.1)	0.27^a^
Delirium, *n* (%)	2 (5.3)	1 (7.1)	1.00^a^
Pruritus, *n* (%)	0 (0)	1 (7.1)	0.27^a^

^a^Fisher’s exact test

**Table 5:** Comparison of the incidence of adverse events between the prophylactic and regular groups in the oxycodone subgroup

Adverse event	Prophylactic oxycodone	Regular oxycodone	*P* value
	*n* = 30	*n* = 21	
Somnolence, *n* (%)	8 (26.7)	3 (14.3)	0.49^a^
Nausea, *n* (%)	5 (16.7)	2 (9.5)	0.69^a^
Constipation, *n* (%)	1 (3.3)	3 (14.3)	0.29^a^
Urinary retention, *n* (%)	1 (3.3)	0 (0)	1.00^a^
Delirium, *n* (%)	1 (3.3)	2 (9.5)	0.56^a^
Pruritus, *n* (%)	0 (0)	0 (0)	1.00^a^

^a^Fisher’s exact test

## Discussion

This study showed no significant difference between the prophylactic and regular rescue dose groups in the incidence of any of the AEs evaluated, including somnolence, nausea, constipation, urinary retention, delirium, and pruritus (Table 3), suggesting that the use of prophylactic rescue dosing and associated increase in total opioid dose do not increase the incidence of AEs. Moreover, with no reported cessation of eating due to nausea, cessation of rehabilitation, intervention or radiation therapy due to somnolence, or refusal of prophylactic rescue dose by patients, it is likely that appropriate prophylactic rescue dosing also improves patients’ QOL. The results of subgroup analysis also suggest the safety of prophylactic rescue dosing regardless of whether morphine syrup or oxycodone powder is used (Tables 4 and 5).

However, comparison of AE incidence between the morphine and oxycodone subgroups showed a lower incidence of constipation in patients using rescue-dose oxycodone, especially in those using prophylactic rescue-dose oxycodone, compared with that in those using rescue-dose morphine. This difference may be explained by the differences in the amount of each rescue single dose and daily total dose between the two subgroups (Table 1). The minimum standard strength of the immediate-release morphine syrup and the immediate-release oxycodone powder in Japan is 5 mg morphine and 2.5 mg oxycodone (3.75 mg morphine equivalence), respectively. The morphine subgroup was associated with a higher frequency of prophylactic rescue doses before meals, with many of these patients having at least 3 doses per day; this might have also contributed to the difference. Another possible explanation is that the patients included in this study were administered relative low doses of extended-release opioids, with a median morphine equivalent dose of 15 mg/day, along with the pharmacological difference that the threshold concentration for causing constipation is lower with morphine than oxycodone [23].

Regarding the purpose of use, patients used prophylactic rescue dosing most commonly before meals in the morphine subgroup and before bedtime in the oxycodone subgroup (Table 2). This may be because many of the patients requiring prophylactic rescue dose before meals experienced pain during swallowing due to underlying disease or prior radiation therapy of the esophagus, and therefore selected the syrup formulation rather than powder, because they are easier to swallow. In contrast, those requiring prophylactic rescue dose before bedtime tended to be concerned about pain during nighttime resting or while falling asleep, and therefore selected oxycodone for the longer-lasting analgesic effect because the half-life is longer than that of morphine [24] (T_1/2_ = 2.2 h for the immediate-release morphine syrup [12] and 6.0 h for the immediate-release oxycodone powder [13]). The higher frequency of use of prophylactic rescue for respiratory distress in the morphine subgroup was probably because evidence for efficacy in the relief of respiratory distress is widely available for morphine [25].

Future prospective studies using a predetermined rescue dose, a dosing frequency, a purpose, a dosing schedule, and a concomitant extended-release agent are needed to accumulate more detailed evidence. Also, we plan to compare other AEs which were unaddressed in the present study and to investigate the efficacy of prophylactic rescue dosing (e.g., by examining decreases in NRS pain assessment results).

Regarding concomitant extended-release opioids, due to the retrospective nature of the study, the presence of patients who used extended-release opioids containing types of opioids different from the immediate-release opioids and those who used no extended-release opioids, might have served as a confounding factor that affected the incidence of AEs. However, given that no serious AE, such as fall, loss of consciousness, respiratory depression, or drug dependence, was observed in the prophylactic group and that no increase in the incidence of somnolence or other AEs was observed in the analysis of the entire population, it is unlikely that serious problems could occur from using a prophylactic rescue dose of an immediate-release opioid with a concomitant extended-release agent containing a different type of opioid or without any concomitant extended-release agent.

## Conclusion

The present results suggest that the incidence of AEs associated with prophylactic rescue dosing is not different from that associated with regular rescue dosing. Moreover, no AE interfered with the objective activity for which the prophylactic rescue dose was used, suggesting the safety of appropriate prophylactic rescue dosing was similar to that of regular rescue dosing.

## Abbreviations

AE: adverse event
CTCAE: Common Terminology Criteria for Adverse Events
EAPC: European Association for Palliative Care
JSPM: Japanese Society for Palliative Medicine
NRS: Numeric Rating Scale
